# miR-145-5p Suppresses Tumor Cell Migration, Invasion and Epithelial to Mesenchymal Transition by Regulating the Sp1/*NF-κB* Signaling Pathway in Esophageal Squamous Cell Carcinoma

**DOI:** 10.3390/ijms18091833

**Published:** 2017-08-23

**Authors:** Li-Li Mei, Wen-Jun Wang, Yun-Tan Qiu, Xiu-Feng Xie, Jie Bai, Zhi-Zhou Shi

**Affiliations:** 1 Medical School, Kunming University of Science and Technology, Kunming 650500, China; 15996663681@163.com (L.-L.M.); 18487182139@163.com (W.-J.W.); yuntan1992@126.com (Y.-T.Q.); 15615741302@163.com (X.-F.X.); 2 State Key Laboratory of Molecular Oncology, Cancer Hospital, CAMS, Beijing 100021, China

**Keywords:** miR-145-5p, esophageal squamous cell carcinoma (ESCC), Sp1, *NF-κB*, epithelial to mesenchymal transition (EMT)

## Abstract

MicroRNAs (miRNAs) play important roles in the progression of human cancer. Although previous reports have shown that miR-145-5p is down-regulated in esophageal squamous cell carcinoma (ESCC), the roles and mechanisms of down-regulation of miR-145-5p in ESCC are still largely unknown. Using microRNA microarray and Gene Expression Omnibus (GEO) datasets, we confirmed that miR-145-5p was down-regulated in ESCC tissues. In vitro assays revealed that ectopic miR-145-5p expression repressed cell proliferation, migration, invasion and epithelial to mesenchymal transition (EMT). miR-145-5p also reduced the expressions of cell cycle genes including cyclin A2 (*CCNA2*), cyclin D1 (*CCND1*) and cyclin E1 (*CCNE1*), the EMT-associated transcription factor Slug, and matrix metalloproteinases (MMPs) including *MMP2*, *MMP7* and *MMP13*. Furthermore, miR-145-5p mimics reduced candidate target gene specificity protein 1 (*Sp1*) and nuclear factor κ B (*NF-κB*) (*p65*) both in mRNA and protein levels. Knockdown of *Sp1* phenocopied the effects of miR-145-5p overexpression on cell cycle regulators, EMT and the expression of *NF-κB* (*p65*). Importantly, inhibition of the *NF-κB* signaling pathway or knockdown of *NF-κB* (*p65*) phenocopied the effects of miR-145-5p on the migration, invasion and EMT of ESCC cells. In conclusion, our results suggested that miR-145-5p plays tumor-suppressive roles by inhibiting esophageal cancer cell migration, invasion and EMT through regulating the *Sp1/NF-κB* signaling pathway.

## 1. Introduction

Esophageal squamous cell carcinoma (ESCC) is a serious health problem in China, with 375,000 new deaths in 2015 alone [1]. Although diagnosis and treatment of ESCC have been improved, the five-year survival rate is still less than 15%. Therefore, understanding of the molecular mechanisms underlying esophageal carcinogenesis will improve the diagnosis and treatment of ESCC.

MicroRNAs (miRNAs) are endogenous, small non-coding RNAs 20–24 nucleotides in length [2]. They bind to the 3′untranslated regions (3′UTRs) of target genes, and regulate the translation and degradation of target mRNAs [3]. miR-145 is significantly down-regulated in ESCC tissues and cell lines compared with normal tissues and cell lines [4,5,6,7], and up-regulation of miR-145 can significantly suppress proliferation, migration, and invasion as well as epithelial–mesenchymal transition (EMT) of ESCC cells by targeting connective tissue growth factor (*CTGF*) [8]. Zhang et al. also reported that overexpression of miR-145 significantly inhibited cell proliferation and induced apoptosis [9]. In ESCC, down-regulation of miR-145 was commonly epigenetically regulated by promoter hypermethylation [10]. However, the tumorigenic roles and mechanisms underlying down-regulation of miR-145 in ESCC are still largely unknown.

In the present study, we found that overexpression of miR-145-5p using mimics inhibited the proliferation, migration, invasion and EMT of esophageal cancer cells. We further found that overexpression of miR-145-5p negatively regulated transcription factor *Sp1* and inhibited the *NF-κB* signaling pathway via decreasing the expression of *NF-κB* (*p65*). Importantly, knockdown of *Sp1* or *NF-κB* (*p65*) and inhibition of the *NF-κB* signaling pathway using CAPE phenocopied the effects of miR-145-5p overexpression on the respective tumor cell phenotypes.

## 2. Results

### 2.1. miR-145-5p Is Down-Regulated in Esophageal Squamous Cell Carcinoma (ESCC) Tissues

**Figure 1:**
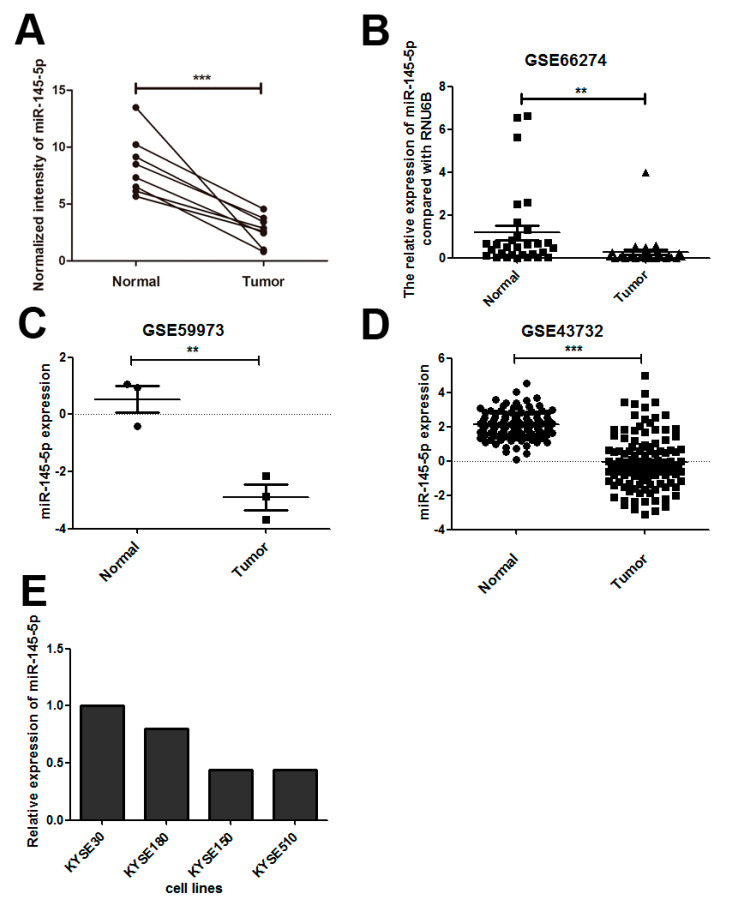
Down-regulation of miR-145-5p in esophageal squamous cell carcinoma (ESCC) tissues. (**A**) Microarray analysis of miR-145-5p expression level in eight paired ESCC tissues and adjacent normal tissues; (**B**–**D**) Low expression of miR-145-5p in ESCC tissues compared with their adjacent non-malignant tissues in the datasets of GSE66274, GSE59973 and GSE43732; (**E**) The expression of miR-145-5p in ESCC cell lines was detected by real-time polymerase chain reaction (RT-PCR). ** *p* < 0.01; *** *p* < 0.001.

We compared the miRNA expression profiles between ESCC tissues and paracancerous tissues using Agilent Human miRNA Microarray, and found that miR-145-5p was down-regulated in ESCC tissues (Figure 1A). We further confirmed our result in three datasets (GSE66274, GSE59973 and GSE43732) of ESCC (Figure 1B–D).

### 2.2. Overexpression of miR-145-5p Inhibits Cell Proliferation, Migration, Invasion and EMT of ESCC Cells

**Figure 2:**
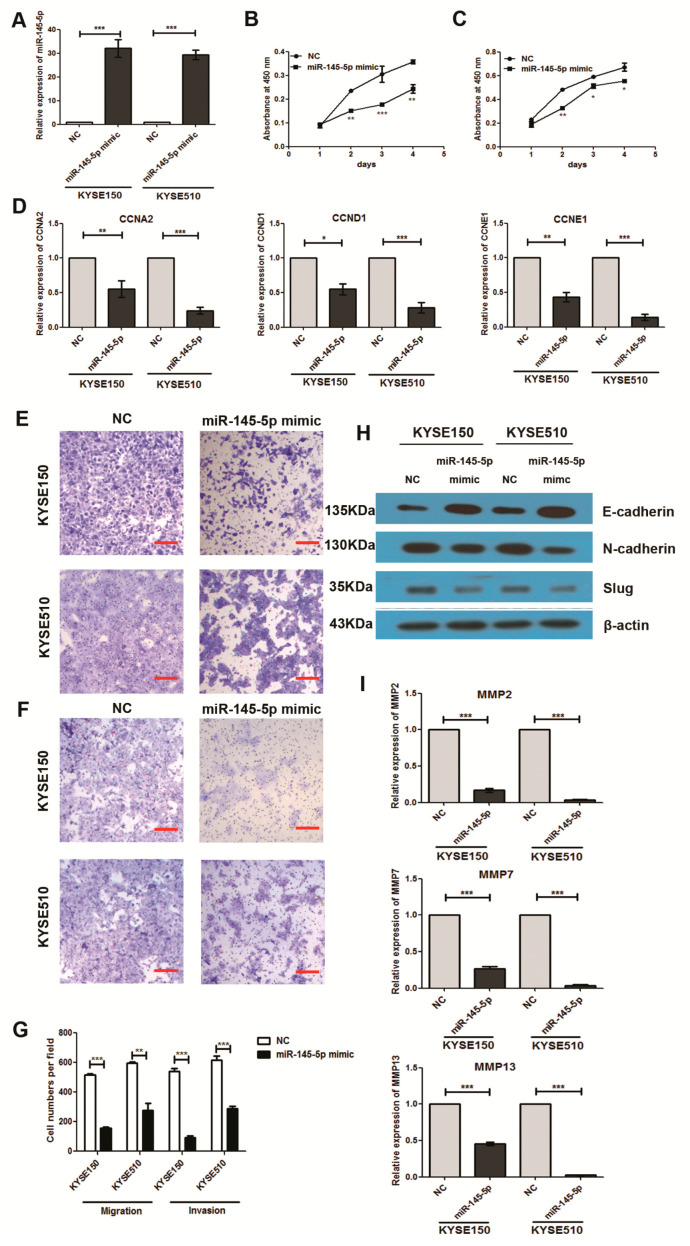
Overexpression of miR-145-5p suppressed ESCC cell proliferation, migration, invasion and epithelial to mesenchymal transition (EMT) of ESCC cells. (**A**) KYSE150 and KYSE510 cells were transfected with miR-145-5p mimics and a non-specific negative control for 48 h. The levels of miR-145-5p were determined by real-time PCR assay (*n* = 3); (**B**,**C**) The cells were treated as indicated above, and the cell proliferation ability was determined by Cell Counting kit-8 (CCK-8) assay; (**D**) The mRNA levels of *CCNA2*, *CCND1* and *CCNE1* were determined by real-time PCR assay (*n* = 3); (**E**–**G**) Cell migration and invasion were detected using Transwell assay. Images of migration and invasion are presented (*n* = 3), the bars represent 200 µm; (**H**) The protein expressions of Slug, E-cadheirn and N-cadherin were measured by Western blotting assay (*n* = 3); (**I**) The mRNA expressions of *MMP2*, *MMP7* and *MMP13* were determined by real-time PCR (*n* = 3). NC: non-specific negative control. Three independent experiments were performed. MMP: matrix metalloproteinase. * *p* < 0.05; ** *p* < 0.01; *** *p* < 0.001.

To study the tumorigenic roles of miR-145-5p in ESCC, we first evaluated the expression levels in four ESCC cell lines including KYSE30, KYSE180, KYSE150 and KYSE510 by real-time polymerase chain reaction (RT-PCR). KYSE150 and KYSE510 exhibited lower expression levels than other two cell lines (Figure 1E). Transfection of miR-145-5p mimics to KYSE150 and KYSE510 cell lines significantly enhanced the expression levels with fold change above 25 compared with negative control group (Figure 2A). Overexpression of miR-145-5p significantly inhibited cell proliferation of KYSE150 and KYSE510 cell lines (Figure 2B,C). *CCNA2*, *CCND1* and *CCNE1* were reported to participate in the regulation of the proliferation process in ESCC [11], and we detected whether miR-145-5p inhibited cell proliferation of ESCC cells via these genes. The results showed that miR-145-5p decreased the mRNA levels of cell cycle regulatory genes (*CCNA2*, *CCND1* and *CCNE1*) in KYSE150 and KYSE510 (Figure 2D). These results suggested that miR-145-5p inhibited the proliferation of ESCC cells via down-regulating *CCND1*, *CCNA2* and *CCNE1*.

Using Transwell assay, we found that miR-145-5p overexpression significantly inhibited the migration and invasion of ESCC cells (Figure 2E–G). Epithelial to mesenchymal transition (EMT) is a major process regulating cell migration and invasion in cancer [12]. Our study further found that overexpression of miR-145-5p significantly increased the epithelial marker of E-cadherin, and reduced the mesenchymal cell marker of N-cadherin (Figure 2H). The protein level of EMT-associated transcription factor Slug was also down-regulated by miR-145-5p mimics (Figure 2H). Our findings suggested that miR-145-5p could significantly repress the migration and invasion of ESCC cells by inhibiting EMT.

*MMP2*, *MMP7* and *MMP13*, which were included in the family of matrix metalloproteinases (MMPs) were reported to play a central role in ESCC cell invasion and metastasis due to their ability to degrade the extracellular matrix [13,14]. Therefore, we detected whether miR-145-5p inhibited cell migration and invasion of ESCC cells via regulating these genes. The results showed that miR-145-5p mimics significantly decreased the expressions of *MMP2*, *MMP7* and *MMP13* using real-time PCR assay (Figure 2I).

### 2.3. Overexpression of miR-145-5p Inhibits the Transcription of Slug via the Sp1/NF-κB Signaling Pathway

**Figure 3:**
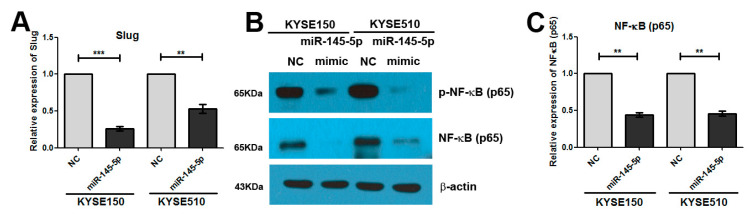
Overexpression of miR-145-5p inhibited the transcription of Slug via the *NF-κB* signaling pathway. (**A**) The mRNA expression of Slug was determined by real-time PCR (*n* = 3); (**B**) The protein levels of *NF-κB* (p65) and p-*NF-κB* (p65) were measured by Western blotting assay (*n* = 3); (**C**) The mRNA expression of *NF-κB* (p65) was determined by real-time PCR (*n* = 3). NC: non-specific negative control. Three independent experiments were performed. ** *p* < 0.01; *** *p* < 0.001.

We also found that miR-145-5p mimics could significantly reduce the mRNA level of Slug (Figure 3A). Therefore, we further explored the mechanism of how miR-145-5p regulates Slug transcription. *NF-κB* is reported to regulate Slug transcription directly [15], and we evaluated whether miR-145-5p mimics inhibited the transcription of Slug via suppression of the *NF-κB* signaling pathway. Our results showed that overexpression of miR-145-5p significantly reduced the levels of *NF-κB* (*p65*) and *p-NF-κB* (*p65*) (Figure 3B), and the mRNA level of *NF-κB* (*p65*) was also decreased (Figure 3C).

**Figure 4:**
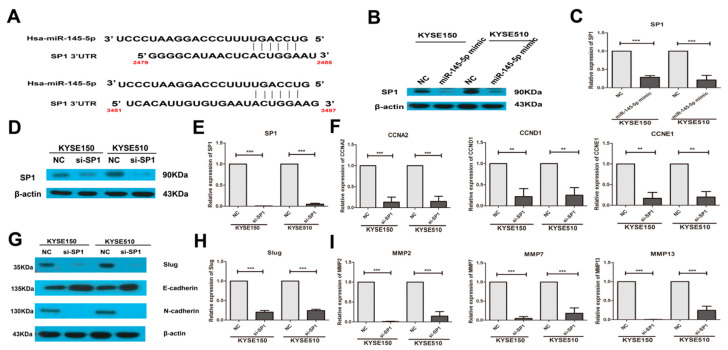
Overexpression of miR-145-5p down-regulated the candidate target gene *Sp1*, and knockdown of *Sp1* phenocopied the effects of miR-145-5p overexpression on cell cycle regulators and EMT. (**A**) The predicted sites of miR-145-5p binding to the 3′untranslated (3′-UTR) region of *Sp1* were detected using bioinformatics prediction tools; (**B**,**C**) KYSE150 and KYSE510 cells were transfected with miR-145-5p mimics or a non-specific negative control for 48 h. The protein and mRNA levels of *Sp1* were measured by Western blotting and real-time PCR assay, respectively (*n* = 3); (**D**,**E**) KYSE150 and KYSE510 cells were transfected with *Sp1* siRNA and a non-specific negative control for 48 h. The protein and mRNA levels of *Sp1* were determined by Western blotting and real-time PCR assay, respectively (*n* = 3); (**F**) The mRNA levels of *CCNA2*, *CCND1* and *CCNE1* were determined by real-time PCR assay (*n* = 3); (**G**) The protein expressions of Slug, E-cadherin and N-cadherin were measured by Western blotting assay (*n* = 3); (**H**) The mRNA levels of Slug were determined by real-time PCR (*n* = 3); (**I**) The mRNA expressions of *MMP2*, *MMP7* and *MMP13* were determined by real-time PCR (*n* = 3). NC: non-specific negative control. Three independent experiments were performed. ** *p* < 0.01; *** *p* < 0.001.

**Figure 5:**
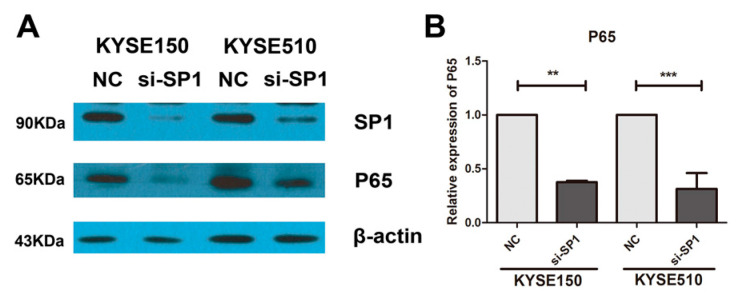
Knockdown of *Sp1* inhibited the transcription of *NF-κB* (*p65*). (**A**) The protein levels of *Sp1* and *NF-κB* (*p65*) were measured by Western blotting assay (*n* = 3); (**B**) The mRNA level of *NF-κB* (*p65*) was determined by real-time PCR assay (*n* = 3). NC: non-specific negative control. Three independent experiments were performed. ** *p* < 0.01; *** *p* < 0.001.

*Sp1* could transcriptionally regulate the expression of *NF-κB* (*p65*), and miR-145-5p could target *Sp1* [16,17,18,19]. Very interestingly, in our study, the results showed that miR-145-5p mimics significantly inhibited the protein and mRNA expressions of *Sp1* (Figure 4A–C). Knockdown of Sp1 in KYSE150 and KYSE 510 cells significantly reduced the levels of *CCNA2*, *CCND1* and *CCNE1* (Figure 4D–F). Silencing of *Sp1* also significantly inhibited the EMT, and down-regulated the expressions of Slug, *MMP2*, *MMP7* and *MMP13* (Figure 4G–I). Importantly, down-regulation of *Sp1* decreased the mRNA and protein levels of *NF-κB* (*p65*) (Figure 5A,B).

### 2.4. Inhibition of NF-κB Signaling Pathway or Knockdown of NF-κB (p65) Phenocopied the Effects of miR-145-5p on the Migration, Invasion and EMT of ESCC Cells

**Figure 6:**
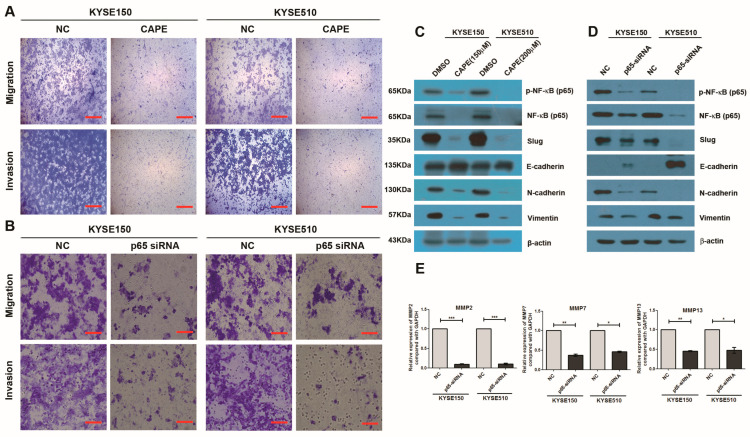
Inhibition of *NF-κB* signaling pathway or knockdown of *NF-κB* (*p65*) phenocopied the effects of miR-145-5p on the migration, invasion and EMT of ESCC cells. (**A**) Cell migration and invasion were detected using Transwell assay under caffeic acid phenethyl ester (CAPE) treatment. Images of migration and invasion are presented (*n* = 3), the bars represent 200 μm; (**B**) Cell migration and invasion were detected using Transwell assay after *p65* small interfering RNA (siRNA) transfection. Images of migration and invasion are presented (*n* = 3), the bars represent 200 μm; (**C**,**D**) Western blotting assay examined the levels of *NF-κB* (*p65*), *p-NF-κB* (*p65*), Slug, E-cadherin, N-cadherin and vimentin (*n* = 3); (**E**) The mRNA expressions of *MMP2*, *MMP7* and *MMP13* were determined by real-time PCR (*n* = 3). NC: non-specific negative control. Three independent experiments were performed. * *p* < 0.05; ** *p* < 0.01; *** *p* < 0.001.

After inhibiting the activity of *NF-κB* signaling pathway using CAPE, the migration and invasion abilities of KYSE150 and KYSE510 cells were decreased (Figure 6A). Knockdown of *NF-κB* (*p65*) also significantly inhibited the migration and invasion of ESCC cells (Figure 6B). CAPE treatment down-regulated the levels of *NF-κB* (*p65*) and *p-NF-κB* (*p65*) in Western blotting assay, and further up-regulated the protein level of E-cadherin, and decreased the levels of N-cadherin, vimentin and Slug (Figure 6C). Knockdown of *NF-κB* (*p65*) also significantly inhibited the EMT by up-regulating the protein level of E-cadherin and decreasing the levels of N-cadherin, vimentin and Slug (Figure 6D). Silencing of *NF-κB* (*p65*) also significantly reduced the mRNA levels of *MMP2*, *MMP7* and *MMP13* (Figure 6E). All of the above suggested that inhibition of the *NF-κB* signaling pathway or knockdown of *NF-κB* (*p65*) phenocopied the effects of miR-145-5p on the migration, invasion and EMT of ESCC cells.

## 3. Discussion

miR-145-5p is down-regulated in many types of cancer including cervical cancer, non-small cell lung cancer (NSCLC), colorectal cancer and ESCC, and functions as tumor suppressor in carcinogenesis [5,8,20,21,22,23]. Metadata showed that the expression of miR-145 was significantly lower in NSCLC than that in healthy tissues, and miR-145 tended to show better diagnostic performance in lung squamous cell carcinoma than in lung adenocarcinoma [24]. In ESCC, the expression of miR-145 was significantly associated with tumor invasion [25]. Combined expression of miR-145 and miR-143 was significantly associated with the risk for ESCC [26].

miR-145 is identified as a tumor suppressor in many types of cancer. In gastric cancer cells, especially cancer stem cells (CSCs), miR-145 regulates CD44 by directly targeting the CD44 3′-untranslated region (3′-UTR) [27]. Overexpression of miR-145 decreases the proliferation, migration and invasion of breast cancer cells via directly or indirectly reducing transforming growth factor-β (*TGF-β*) expression [28]. In bladder cancer cells, miR-145 is down-regulated and its mimics reduce the Warburg effect by directly silencing kruppel like factor 4 (*KLF4*) [29]. miR-145 inhibits drug resistance to oxaliplatin in colorectal cancer cells by suppressing the expression level of target gene *GPR98* [30]. In ESCC, miR-145 is commonly downregulated, and overexpression of miR-145 in esophageal ECA109 and EC9706 cells could inhibit cell proliferation and induce apoptosis [9]. Wang et al. reported that overexpression of miR-145 using the pLVX-IZ-miR-145 vector significantly inhibited esophageal cancer cell ECA109 proliferation, and increased the number of cells at the G2/M stage and the cell apoptotic rate [31]. Wang et al. further found that miR-145 inhibited the proliferation and invasion of ESCC ECA109 and EC9706 cells in part by targeting c-Myc [6]. Cui et al. found that miR-145 directly targeted the 3′UTR of phospholipase C epsilon (*PLCE1*) and then inhibited cell proliferation, migration and metastasis, as well as controlling the cytoskeleton dynamics of esophageal cancer [4]. Han et al. reported that miR-145 could bind with the 3′UTR of connective tissue growth factor (*CTGF*) in dual luciferase reporter gene assay, and up-regulation of miR-145 or down-regulation of *CTGF* could suppress the proliferation, migration, invasion and EMT process of ESCC ECA109 cells [8]. Previous studies also reported that overexpression of miR-145 could reduce target gene fascin-1 (*FSCN1*) expression [26,32]. All these reports indicated that miR-145 regulated the proliferation and invasion of ESCC cells through several target genes and signaling pathways. Our results confirmed that miR-145-5p was down-regulated in ESCC tissues, and overexpression of miR-145-5p inhibited cell proliferation, migration, invasion and EMT of ESCC cells. Very importantly, we further found that overexpression of miR-145-5p inhibited the transcription of EMT associated transcription factor Slug via suppression of the *Sp1*/*NF-κB* signaling pathway. Inhibition of *NF-κB* signaling pathway or knockdown of *NF-κB* (*p65*) phenocopied the effects of miR-145-5p on the migration, invasion and EMT of ESCC cells. Taken together, our results revealed the new signaling pathway regulated by miR-145 in esophageal carcinogenesis.

miR-145 is regulated by several mechanisms in cancer. The miR-145 promoter is significantly more hypermethylated in ESCC cancer tissues than in matched normal adjacent esophageal epithelial mucosa [10]. Li et al. found that ginsenoside 20(S)-Rg3, a pharmacologically active component of Panax ginseng, could increase miR-145 expression by downregulating methyltransferase DNMT3A to attenuate the hypermethylation of the promoter region in the miR-145 precursor gene [33]. LincRNA is also a very important regulator of miR-145 in cancers. Low expression of miR-145 is associated with poor prognosis in NSCLC, and silencing lincRNA ROR posttranscriptionally regulates the expression of p53, while silencing ROR or p53 could upregulate miR-145 levels [21]. LincRNA ROR could also sponge miR-145 and then release the miR-145 target FSCN1, and further contribute to the acquisition of chemoresistance and EMT phenotypes of docetaxel-resistant lung adenocarcinoma cells [34]. LincRNA MALAT1 could modulate the *TGF-β1*-induced endothelial-to-mesenchymal transition through interacting and down-regulating miR-145 [35]. LincRNA CRNDE was highly expressed in gastric cancer cells, and overexpression of *CRNDE* increased cell viability and promoted colony formation via sponging miR-145 and activation of its target gene *E2F3* [36]. However, up to now, the mechanisms underlying down-regulation of miR-145 in ESCC are still largely unknown.

Taken together, our findings revealed that miR-145-5p functioned as a tumor suppressor gene by regulating the *Sp1*/*NF-κB* signaling pathway in esophageal squamous cell carcinoma. Future studies should focus on the diagnostic and prognostic value of miR-145 in ESCC.

## 4. Materials and Methods

### 4.1. Sample Information and miRNA Expression Microarray Detection

Fresh tissues of ESCC and adjacent normal epithelia from eight patients with ESCC were collected by the Department of Pathology at the Cancer Hospital, Chinese Academy of Medical Sciences (Beijing, China). Primary tumor tissues and the adjacent normal tissues from the same patients were separated by experienced pathologists and then immediately stored at −70 °C. All ESCC patients were treated with radical operation, and none of them received any preoperative treatment. All the samples were residual specimens after diagnostic sampling, and all patients signed separate informed consent forms for sampling and molecular analysis. This was approved by the Ethics Committee of Kunming University of Science and Technology (No. 2014KGGJLL005).

Agilent Human miRNA Microarrays (Agilent Technologies, Santa Clara, CA, US) were used to examine miRNA expression levels in paired tumorous and paracancerous tissues. The microarray experiment was performed according to the manufacturer’s protocol. The raw data were normalized using quantile normalization and then analyzed in GeneSping GX 11.0 software (Agilent Technologies, Santa Clara, CA, USA).

### 4.2. Gene Expression Omnibus (GEO) Datasets

GSE66274, GSE59973 and GSE43732 were accessible at the National Center of Biotechnology Information (NCBI) Gene Expression Omnibus database (http://www.ncbi.nlm.nih.gov/geo/). GSE66274 was based on the GPL19823 platform of the Applied Biosystems Taqman Low Density Array Human microRNA Card A+B Set v3.0. A total of 60 chips were divided into two groups: ESCC tissue samples (*n* = 30) and normal samples (*n* = 30). GSE59973 was based on the GPL16770 platform of the Agilent-031181 Unrestricted_Human_miRNA_V16.0_Microarray (miRBase release 16.0 miRNA ID version). A total of six chips were divided into two groups: ESCC tissue samples (*n* = 3) and normal samples (*n* = 3). GSE43732 was based on the GPL16543 platform of Agilent-038166 cbc_human_miR18.0. A total of 238 chips were divided into two groups: ESCC tissue samples (*n* = 119) and adjacent normal tissue samples (*n* = 119).

### 4.3. Cell Culture

The human esophageal squamous cell carcinoma (ESCC) cell lines, including KYSE30, KYSE150, KYSE180 and KYSE510, were provided by Yutaka. Shimada (Kyoto University, Kyoto, Japan). The cell lines were cultured in RPMI-1640 medium (Invitrogen, Carlsbad, CA, USA) with 10% fetal bovine serum, penicillin (100 U/mL) and streptomycin (100 mg/mL). All of these cells were maintained at 37 °C with 5% CO_2_.

### 4.4. Cell Transfection

The cells were seeded in six-well plates and transfected with miR-145-5p mimics or *Sp1* small interfering RNA (siRNA) or *NF-κB* (*p65*) siRNA or non-specific negative control using Lipofectamine 2000 Transfection Reagent (Invitrogen, Carlsbad, CA, USA) following the manufacture’s protocol. miR-145-5p mimics sense: 5′-GUCCAGUUUUCCCAGGAAUCCCU-3′, antisense: 5′-GGAUUCCUGGGAAAACUGGACUU-3′; non-specific negative control sense: 5′-UUCUCCGAACGUGUCACGUTT-3′, antisense: 5′-ACGUGACACGUUCGGAGAATT-3′; *Sp1* siRNA sense: 5′-UGAGAACAGCAACAACUCCTT-3′, antisense 5′-GGAGUUGUUGCUGUUCUCATT-3′; *p65* siRNA sense: 5′-GCCUUAAUAGUAGGGUAAGTT-3′, antisense: 5′-CUUACCCUACUAUUAAGGCTT-3′. miR-145-5p mimics, *Sp1* siRNA, *NF-κB* (*p65*) siRNA and non-specific negative control were synthesized by GenePharma (GenePharma, Shanghai, China).

### 4.5. Cell Proliferation Assay

Cellular proliferation was measured by Cell Counting Kit-8 (CCK-8, Dojindo Laboratories, Kumamoto, Japan) based on the manufacturer’s instructions. Twenty-four hours after transfection, cells were seeded into 96-well plates at a density of 8 × 10^3^ cells per well with 100 µL of cell culture medium and incubated with 5% CO_2_ at 37 °C. At the indicated time points, 10 µL CCK-8 was added to each well. After incubation for 1 h, the absorbance at 450 nm was detected by a plate reader.

### 4.6. Cell Migration and Invasion Assay

Transwell assay was used to analyze the migration and invasion abilities of KYSE150 and KYSE510 cells. In the invasion assay, the upper side of the membrane was pre-coated with Matrigel (BD Biosciences, San Jose, CA, USA), incubated for 1 h at 37 °C, and hydrated in fetal bovine serum (FBS) for two hours before use. The cells were digested and seeded into the upper chamber at the cell density of 3 × 10^5^ cells/mL. The lower chamber was added with cell culture medium containing 20% FBS, and incubated at 37 °C for 36 h. After incubation, 0.1% crystal violet was used to stain the membrane for 30 min. After washing by Phosphate Buffered Saline (PBS), stained cells were counted using an optical microscope. For cell migration assay, the same procedures were conducted but without Matrigel on the membrane.

### 4.7. Total RNA Extraction and Real-Time PCR Assay

Total RNA was isolated from cancer cells using the RNeasy Mini Kit as described by the manufacturer (Qiagen, Hilden, Germany) and used for real-time PCR assay.

Real-time PCR was performed to detect the relative expression levels of *CCNA2*, *CCND1*, *CCNE1*, *MMP2*, *MMP7*, *MMP13*, *Sp1* and *p65*. The PCR reactions were performed in a total volume of 20 µL, including 10 µL of 2 × Power SYBR ® Green PCR Master Mix (Applied Biosystems, Warrington, UK), 2 µL of cDNA (5 ng/µL) and 1 µL of primer mix (10 µM each). PCR amplification was performed using the LightCycler 480 II (Roche Applied Science, Penzberg, Germany). The program of PCR amplification was as follows: 10 min of denaturation at 95 °C; 40 cycles of 95 °C for 15 s, and then 60 °C for 1 min. The relative gene expression level was calculated by using the comparative *C*t Method. The gene expressions of the detected genes were normalized to an endogenous reference glyceraldehyde-3-phosphate dehydrogenase (*GAPDH*), and those relative to the calibrator were given by the formula 2^−∆∆*C*t^.

The Hairpin-itTM miR-145 qRT-PCR Primer Set (GenePharma, Shanghai, China) was used for the measurement of the relative quantity of miR-145-5p. U6 was used as an internal control, and the mRNA expression of miR-145-5p was normalized to the endogenous expression of U6.

### 4.8. Western Blotting Assay

Cells from each group were detached with trypsin, centrifuged, and washed two times with pre-chilled PBS. Cell lysis buffer was subsequently added and incubated on ice for protein extraction. Protein concentration was determined using the BCA Protein Assay Kit (Beyotime Biotechnology, Jiangsu, China). Equal amounts of proteins were separated via 12% sodium dodecyl sulfate polyacrylamide gel electrophoresis (SDS-PAGE) and then transferred to a polyvinylidene fluoride (PVDF) membrane (Millipore Corporation, Billerica, MA, USA). The membrane was soaked in 10% skimmed milk (in PBS, pH 7.2, containing 0.1% Tween-20) for 2 h and incubated with an appropriate amount of primary antibody at 4 °C overnight. Detection was done by peroxidase-conjugated secondary antibodies (KPL, Gaithersburg, MD, USA) and chemiluminescence (Milipore Corporation, Temecula, CA, USA).

### 4.9. Statistical Analyses

All quantitative data are presented as mean ± standard deviation (SD). Student’s *t* test was performed when only two groups were present using GraphPad Prism version 5.01 (GraphPad Software, La Jolla, CA, USA). The differences were judged as statistically significant when the corresponding two-sided *p* value was <0.05.
