# Correction: Mei et al. miR-145-5p Suppresses Tumor Cell Migration, Invasion and Epithelial to Mesenchymal Transition by Regulating the Sp1/*NF-κB* Signaling Pathway in Esophageal Squamous Cell Carcinoma. *Int. J. Mol. Sci.* 2017, *18*, 1833

**DOI:** 10.3390/ijms27125247

**Published:** 2026-06-10

**Authors:** Li-Li Mei, Wen-Jun Wang, Yun-Tan Qiu, Xiu-Feng Xie, Jie Bai, Zhi-Zhou Shi

**Affiliations:** 1 Medical School, Kunming University of Science and Technology, Kunming 650500, China; 15996663681@163.com (L.-L.M.); 18487182139@163.com (W.-J.W.); yuntan1992@126.com (Y.-T.Q.); 15615741302@163.com (X.-F.X.); 2 State Key Laboratory of Molecular Oncology, Cancer Hospital, Chinese Academy of Medical Sciences, Beijing 100021, China

In the original publication [1], there was a mistake in Figure 6A as published. An error occurred during the selection and arrangement processes of the images. The corrected Figure 6 appears below. The authors state that the scientific conclusions are unaffected. This correction was approved by the Academic Editor. The original publication has also been updated.

## Figures and Tables

**Figure 6:**
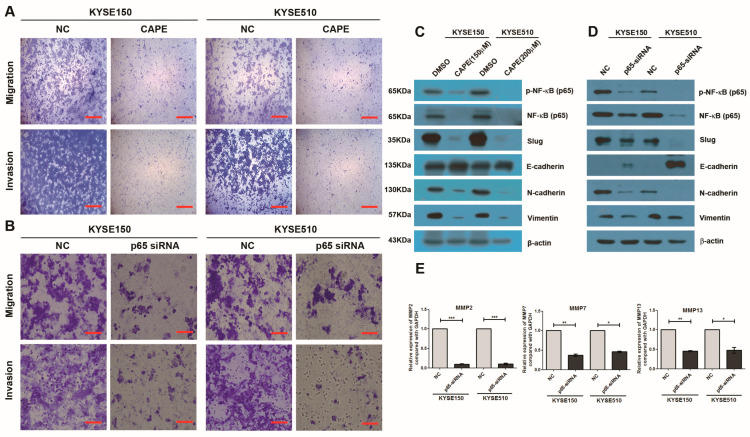
Inhibition of *NF-κB* signaling pathway or knockdown of *NF-κB* (*p65*) phenocopied the effects of miR-145-5p on the migration, invasion and EMT of ESCC cells. (**A**) Cell migration and invasion were detected using Transwell assay under caffeic acid phenethyl ester (CAPE) treatment. Images of migration and invasion are presented (*n* = 3), the bars represent 200 μm; (**B**) Cell migration and invasion were detected using Transwell assay after *p65* small interfering RNA (siRNA) transfection. Images of migration and invasion are presented (*n* = 3), the bars represent 200 μm; (**C**,**D**) Western blotting assay examined the levels of *NF-κB* (*p65*), *p-NF-κB* (*p65*), Slug, E-cadherin, N-cadherin and vimentin (*n* = 3); (**E**) The mRNA expressions of *MMP2*, *MMP7* and *MMP13* were determined by real-time PCR (*n* = 3). NC: non-specific negative control. Three independent experiments were performed. * *p* < 0.05; ** *p* < 0.01; *** *p* < 0.001.
